# Modeling of Aerosol Vertical Profiles Using GIS and Remote Sensing

**DOI:** 10.3390/s90604380

**Published:** 2009-06-04

**Authors:** Man Sing Wong, Janet E. Nichol, Kwon Ho Lee

**Affiliations:** 1 Department of Land Surveying and Geo-Informatics, The Hong Kong Polytechnic University, Hong Kong, China; E-Mail: m.wong06@fulbrightmail.org; 2 Department of Land Surveying and Geo-Informatics, The Hong Kong Polytechnic University, Hong Kong, China; 3 Earth System Science Interdisciplinary Center, University of Maryland, College Park, MD 20740, USA; E-Mail: kwonlee@umd.edu

**Keywords:** aerosol optical thickness, extinction coefficient, GIS, modeling, visualization

## Abstract

The use of Geographic Information Systems (GIS) and Remote Sensing (RS) by climatologists, environmentalists and urban planners for three dimensional modeling and visualization of the landscape is well established. However no previous study has implemented these techniques for 3D modeling of atmospheric aerosols because air quality data is traditionally measured at ground points, or from satellite images, with no vertical dimension. This study presents a prototype for modeling and visualizing aerosol vertical profiles over a 3D urban landscape in Hong Kong. The method uses a newly developed technique for the derivation of aerosol vertical profiles from AERONET sunphotometer measurements and surface visibility data, and links these to a 3D urban model. This permits automated modeling and visualization of aerosol concentrations at different atmospheric levels over the urban landscape in near-real time. Since the GIS platform permits presentation of the aerosol vertical distribution in 3D, it can be related to the built environment of the city. Examples are given of the applications of the model, including diagnosis of the relative contribution of vehicle emissions to pollution levels in the city, based on increased near-surface concentrations around weekday rush-hour times. The ability to model changes in air quality and visibility from ground level to the top of tall buildings is also demonstrated, and this has implications for energy use and environmental policies for the tall mega-cities of the future.

## 1. Introduction

Estimating the atmospheric aerosol content is important in both remote sensing and climatology [1,2]. Although satellite aerosol retrieval algorithms are well-developed, they are only used to map aerosols as they vary over horizontal space, while vertical aerosol profiles still rely on “*in-situ*” and active remote sensing data (e.g. from aircraft, balloons, and Lidar). However, aerosol vertical profiles are important for understanding the radiative effects of aerosols and for generating more accurate aerosol models than those from passive remote sensing [3,4], which estimate aerosol amounts for the whole atmospheric column as a unitless measure, Aerosol Optical Thickness (AOT, τ). While Lidar measurements do provide data for different atmospheric layers and height dependent extinction values [5-7], the high instrument cost and its complicated installation prohibit its widespread use. Although aerosol profiles cannot be obtained directly from ground-based chemical and physical measurements, recent work [8-11] demonstrates that they can be derived by ground-based sensing of the properties of the atmospheric column using a multi-channel sunphotometer. These estimations are based on the computation of aerosol scaling height from surface visibility data as well as the columnar aerosol properties from the sunphotometer. Visualization of the aerosol vertical profiles can then be implemented in a Geographic Information System-based aerosol model. The technical basis and theory relating to such a model are discussed below.

Generally, the aerosol vertical profile when remotely sensed from the ground, is expressed as an extinction coefficient σ, that is, the fraction of light lost to scattering and absorption by aerosol particles as a function of altitude. The aerosol extinction coefficient at surface level can be derived from the visibility (or “visual range”). Since the integrated extinction coefficient over a vertical column of unit cross section corresponds to AOT, the aerosol extinction at each altitude can be calculated using a known AOT value and aerosol scaling height. The aerosol scaling height is a measure of the decrease of aerosol loadings over an altitude, and can be estimated from the surface visibility and AOT value. For visualization of the atmospheric conditions at different elevations, the extinction coefficients can be converted to the Δτ (AOT values at different atmospheric levels) by multiplying the columnar AOT with the extinction coefficient at different elevations. This is a better way of analyzing urban aerosols than using a whole column aerosol measure such as AOT.

A Geographic Information System provides a platform for integrating diverse data types including both raster and vector in 2-, 3- [11-15], and more recently 3.5-dimensions [16]. In Hong Kong, the Hong Kong Observatory has adopted a GIS platform to handle large volumes of meteorological and geophysical data in a spatial framework for weather reporting [17]. However there has been no attempt in Hong Kong or elsewhere to use GIS for analysis and visualization of vertical aerosol profiles. This study demonstrates a near-real time methodology for visualizing aerosol vertical profiles over an urban area, by integrating them with spatially referenced terrain elevation and building height data on a GIS platform. Surface visibility is an important factor in determining near-surface aerosol loadings, and based on this, Elterman [8] devised a vertical attenuation model for the near surface layer, with an assumed aerosol top layer at 5 km which was highly dependent on visibility values. In this paper, a more empirical model after Qiu [9] and Qiu *et al.* [10] is used, which measures the atmospheric profile to estimate scale heights and thus derive aerosol loadings at different vertical heights.

We adapted the algorithms developed by Qiu [9] and Qiu *et al.* [10] which estimate the extinction coefficient and scaling height from a pyrheliometer, for use with an AERONET sunphotometer. Two of these have been established in Hong Kong, in collaboration with NASA. Qiu *et al.* [10] found a low absolute standard error of the extinction coefficient (i.e. 0.0023 km^-1^) for background aerosols when the pyrheliometer aerosol profiles were compared with those calculated from MODTRAN. The accuracy of computing these from AERONET was tested by Wong *et al.* [11] using data of the year 2006 from Taipei where an AERONET and co-located MPLNET Lidar are available. Cloud-screening and temporal matching were undertaken, and only matched data (within 30 minutes) were selected for analysis. A total of 164 matched AERONET and MPLNET data were found. A low error of the extinction coefficient of 0.004 km^-1^ was observed for a normal urban atmosphere in Taipei. These compare with much higher absolute standard errors for conditions following a strong, and an extremely strong volcano, of 0.0097 and 0.0316 km^-1^ respectively [10]. Thus it may be assumed that for a normal aerosol profile under non-dust and cloud-free atmospheric conditions (i.e. typical of Hong Kong under normal conditions), the extinction coefficient derived in section 3.1 is acceptable.

In this study, a GIS is used to display the aerosol vertical distribution in 3D, for visualization in context of the city's built environment.

## 2. Data Collection

The study area corresponds to the densely urbanized Kowloon peninsula of Hong Kong which contains an AERONET, and a meteorological station, both located near the centre of the peninsula. The AERONET is a multi-channel Cimel sunphotometer which senses aerosols upward through the atmospheric column and thus derives AOT every 15 minutes during daylight hours, and is connected to NASA's Aerosol Robotic NETwork [18]. The AERONET level 1.5 cloud-screened AOT data [19,20] at 440nm, 675nm and Angstrom exponent are used for computing AOT at 550 nm, and this is used for subsequent calculation of extinction profiles and scaling heights. Surface visibility data were acquired from the Hong Kong Observatory which is only 500 m from the AERONET site, the paired AOT and visibility readings were within a 30 minutes time difference. A study of long-term visibility in Hong Kong reported that visibility differences between west (Hong Kong International airport) and central (Hong Kong Observatory) Hong Kong have a range of 0.02 to 2.64 km [21], but these two stations are 27 km away from each other. Therefore, visibility in small area corresponding to Hong Kong urban area is assumed to vary insignificantly and the surface visibility data from the Hong Kong Observatory can be used to represent for Hong Kong urban area.

## 3. Methodology

### 3.1. Modeling the Aerosol Vertical Profiles Using AERONET and Climatological Data

The aerosol scaling height (z_a_) for the modeling of aerosol extinction profile (*σ_a_*(*z*)) is defined as the height of an exponential profile at which the value is decreased to 1/e of the value at ground level (*σ_a_*(*z*=0)):

(1) $$\sigma_{a}\left(z\right)=\sigma_{a}\left(z=0\right)\exp\left[-z/z_{a}\right]$$

Aerosol optical thickness (τ_a_), the integral with heights to the aerosol extinction, can be written as:

(2) $$\tau_{a}=\int_{0}^{\text{TOA}}\sigma_{a}\left(z\right)dz$$

(3) $$=\sigma_{z}\left(z=0\right)\cdot z_{a}\left[1‐\exp\left(‐z_{\text{TOA}}/z_{a}\right)\right]$$

The exponential term in equation (3) is negligible because typical z_a_ values are less than 2km in China [10] while it is sometimes higher in other places [22]. *σ_a_*(*z*=0) has been found to be inversely proportional to the visibility range (Vis), and can be estimated by the empirical Koschmieder equation [23,24] as well. Then z_a_ can be derived as:

(4) $$z_{a}=\frac{\tau_{a}}{\sigma_{a}}=\frac{\tau_{a}}{3.912/\text{Vis}-\sigma_{m}\left(z=0\right)}$$

where σ_m_ is the surface-level molecular extinction coefficient. The assumption made in the model was that the extinctions produced by the background stratospheric aerosol and gases at 550 nm are negligible, compared with those from the tropospheric aerosols. Qiu *et al.* [9] showed that the absolute standard error of extinction coefficient from the aerosol scale height is 0.0023 km^-1^ in the case of background aerosols, and that good consistency exists between calculated and MODTRAN aerosol profile under non-dust and cloud-free atmospheric conditions. In order to derive z_a_ and τ_a_(z), the AERONET level 1.5 AOT at 550 nm data (τ_a_), and surface visibility (Vis) data were used in this study.

### 3.2. Linkage with GIS Platform

**Figure 1.**
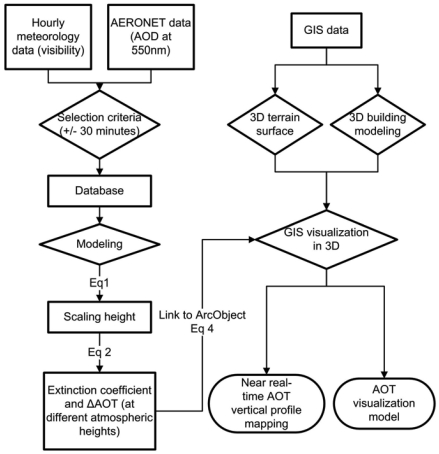
Flowchart for modeling aerosol vertical profiles on a GIS platform.

This study is the first to compute atmospheric extinction coefficients from AERONET, instead of from the more costly MPLNET, and the first to demonstrate a method for near real-time AOT vertical profile mapping and visualization. Figure 1 illustrates the workflow.

The computation of the near-real time vertical profiles are done by a digital link-up between AERONET and ESRI® ArcGIS™ 9.2 software, with automated download from the sunphotometer, and simultaneous upload to NASA website and to our GIS server. A customized script written in ArcObject in ArcGIS links the visibility to the AOT data, and calculates the scaling height, extinction vertical profiles and Δτ based on the columnar aerosol properties and ground-level visibility (Equation 5):

(5) $$\Delta \tau =\Delta \sigma_{z}\cdot \Delta z$$

**Figure 2.**
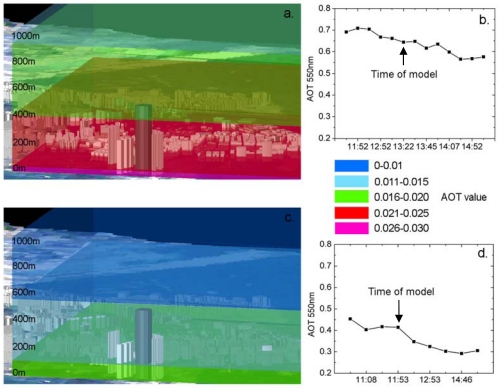
Derived AOT for different atmospheric layers, 3D view from across Victoria harbour to the high rise buildings on Kowloon peninsula on a. 01-Feb-2007 (Local time 01:22 p.m.), c. 03-Feb-2007 (Local time 11:53 a.m.). The graphs (b and d) represent processed level 1.5 AERONET AOT data for 550 nm collected over the course of the day.

Once the Δτ is calculated, the scripts are written to access the functionality in ArcScene for polygon extrusion and format conversion [25]. The variables for adjusting the column attributes in the GIS in near-real time are derived for the visualization. This is done by a link between the ArcScene and the vertical profile database. The scripts include the predefinition of the aerosol layers (aerosol polygons at constant intervals of 75 m), and their linkage with the database, as well as their colour assignment (six transparent shades were used to represent AOT concentrations at different levels (Figures 2a and 2c). The geo-referenced terrain elevations with building heights in the high rise urban landscape are constructed using digital data of building and road polygons from the Lands Department. The scale of the model was set to 1:5,000 and it was linked to aerosol data. The key elements of the coding written in ArcObject can be accessed in http://www.lsgi.polyu.edu.hk/rsrg/resources/pj/UHI/3d_coding.txt.

## 4. Results

Two days in February 2007 were selected for demonstrating the model visual outputs. On 01-Feb-2007, a weekday with low wind speeds, the AERONET data (Figure 2b) shows high urban pollutant levels (AOT 0.56 to 0.71). The image vertical profile for 1 p.m. on this day (Figure 2a) indicates that pollutants are especially concentrated below 100 m. This strongly suggests a local source such as vehicle emissions. The evident decrease in aerosol concentration with height within the urban canopy layer, indicating better visibility and better air quality on higher building floors, gives the option to open windows for indoor ventilation.

However, on 03-Feb-2007, a weekend, and with higher wind speeds, the AERONET data (Figure 2d) shows only moderate pollutant levels (AOT 0.3 to 0.5). The image vertical profile at midday (Figure 2c), shows surface level concentrations to be relatively low (0.015 to 0.02), and very low near the mountain tops. Table 1 shows the AOT values, visibilities and scaling heights for both days.

**Figure 3.**
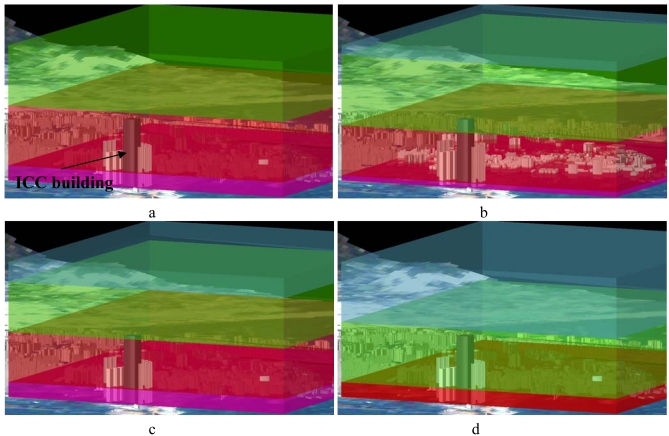
Time series of ΔAOT over the Kowloon peninsula, on 01-Feb-2007 at local times a. 11:52 a.m., b. 12:52 p.m., c. 01:22 p.m., d. 03:07 p.m., overlaid onto a 3D model of the urban landscape. The 500 m tall International Commerce Centre is indicated at bottom left.

Figure 3 demonstrates the near real-time capability of the model, with a time series of AOT profiles from 11:52 a.m. to 03:07 p.m. on 01-Feb-2007. The Δτ at surface level was particularly high between 11:52 a.m. and 01:22 p.m., possibly due to the peak traffic around lunch time, which again suggests that vehicle emissions were the dominant source of aerosol in urban areas on that date. The International Commerce Centre (ICC) which, at 500 m is the world's third tallest building can be seen in the bottom left corner of Figure 3. The figure suggests that heavy pollutants earlier in the day had largely dispersed by 3 p.m. and all floors upwards of 75 m had relatively good visibility and air quality.

The scaling heights calculated for these cases are less than 2 km, which is similar to the findings of Qiu *et al.* [10] (Table 1) and visibilities of 9 to 10 km were observed. Since this method only can be applied whenever AERONET data and visibility data are coincident under clear sky condition, only 56% (2360/4209) of the data collected for our study can be used for modeling, with the other 44% being cloudy.

## 5. Discussion and Conclusions

The study presents a state-of-the-art technique for modeling and visualization of atmospheric vertical profiles using AERONET level 1.5 AOT and surface visibility data. The AOT values for different atmospheric heights were linked to a GIS-based 3D urban model to provide near-real time visualization over the 3D urban landscape. These processes permit analysis of aerosol concentrations at near surface levels, which may help to shape policy decisions on environmental protection. Linkage to a health hazard warning system would be a useful option since increased vertical extent in the world's developing mega-cities will be necessary for efficiency and energy conservation.

This technique can be used in any city where there is a local AERONET and climatic station, which can supply both near-real time AOT and visibility data, and environmental authorities may easily use the method by having the GIS software and a semi-specialist GIS programmer. The model could be further extended to give horizontal, as well as vertical AOT variations, by linkage to MODerate resolution Imaging Spectroradiometer (MODIS) aerosol images. However, the temporal resolution would decrease from near-real time to daily, and the horizontal resolution would increase to 500 m, which is the best resolution currently available for AOT retrieval from MODIS images [26,27]. This prototype will be developed with user-interface query and web-interface systems in the near future.

## Figures and Tables

**Table 1.** Summary of AOT value, visibility and scaling height.

**Date**	**Time**	**AOT at 550 nm**	**Visibility (km)**	**Scaling height z_a_ (km)**

01-Feb-2007	11:52 a.m.	0.71	10	1.81
01-Feb-2007	12:52 a.m.	0.67	10	1.71
01-Feb-2007	01:22 p.m.	0.64	9	1.48
01-Feb-2007	03:07 p.m.	0.58	10	1.47
03-Feb-2007	11:53 a.m.	0.41	9	0.95
